# Type 1 diabetes in Africa: an immunogenetic study in the Amhara of North-West Ethiopia

**DOI:** 10.1007/s00125-020-05229-x

**Published:** 2020-07-23

**Authors:** Shitaye A. Balcha, Abayneh G. Demisse, Rajashree Mishra, Tanwi Vartak, Diana L. Cousminer, Kenyaita M. Hodge, Benjamin F. Voight, Kim Lorenz, Stanley Schwartz, Samuel T. Jerram, Arla Gamper, Alice Holmes, Hannah F. Wilson, Alistair J. K. Williams, Struan F. A. Grant, R. David Leslie, David I. W. Phillips, Elisabeth R. Trimble

**Affiliations:** 1 Department of Internal Medicine, Gondar University Hospital, Gondar, Ethiopia; 2 Department of Pediatrics and Child Health, School of Medicine, University of Gondar, Gondar, Ethiopia; 3 Division of Human Genetics, The Children’s Hospital of Philadelphia, Philadelphia, PA USA; 4 Graduate Group in Genomics and Computational Biology, Perelman School of Medicine, University of Pennsylvania, Philadelphia, PA USA; 5 Center for Spatial and Functional Genomics, The Children’s Hospital of Philadelphia, Philadelphia, PA USA; 6 Blizard Institute, Queen Mary University of London, London, UK; 7 Department of Genetics, Perelman School of Medicine, University of Pennsylvania, Philadelphia, PA USA; 8 Department of Systems Pharmacology and Translational Therapeutics, Perelman School of Medicine, University of Pennsylvania, Philadelphia, PA USA; 9 Main Line Health System, Wynnewood, PA USA; 10 Severn Postgraduate School of Primary Care, Health Education England, Bristol, UK; 11 Avon and Wiltshire Mental Health Partnership NHS Trust, Clevedon, UK; 12 Diabetes and Metabolism, Translational Health Sciences, University of Bristol, Southmead Hospital, Bristol, UK; 13 Institute for Diabetes, Obesity and Metabolism, Perelman School of Medicine, University of Pennsylvania, Philadelphia, PA USA; 14 Department of Pediatrics, Perelman School of Medicine, University of Pennsylvania, Philadelphia, PA USA; 15 MRC Lifecourse Epidemiology Unit, University of Southampton, Southampton General Hospital, Southampton, UK; 16 Centre for Public Health, Institute of Clinical Science, Queen’s University Belfast, Grosvenor Road, Belfast, BT12 6BA UK

**Keywords:** Africa, Autoantibodies, Ethiopia, Genomes, HLA, Rural, Type 1 diabetes

## Abstract

**Aims/hypothesis:**

We aimed to characterise the immunogenic background of insulin-dependent diabetes in a resource-poor rural African community. The study was initiated because reports of low autoantibody prevalence and phenotypic differences from European-origin cases with type 1 diabetes have raised doubts as to the role of autoimmunity in this and similar populations.

**Methods:**

A study of consecutive, unselected cases of recently diagnosed, insulin-dependent diabetes (*n* = 236, ≤35 years) and control participants (*n* = 200) was carried out in the ethnic Amhara of rural North-West Ethiopia. We assessed their demographic and socioeconomic characteristics, and measured non-fasting C-peptide, diabetes-associated autoantibodies and *HLA-DRB1* alleles. Leveraging genome-wide genotyping, we performed both a principal component analysis and, given the relatively modest sample size, a provisional genome-wide association study. Type 1 diabetes genetic risk scores were calculated to compare their genetic background with known European type 1 diabetes determinants.

**Results:**

Patients presented with stunted growth and low BMI, and were insulin sensitive; only 15.3% had diabetes onset at ≤15 years. C-peptide levels were low but not absent. With clinical diabetes onset at ≤15, 16–25 and 26–35 years, 86.1%, 59.7% and 50.0% were autoantibody positive, respectively. Most had autoantibodies to GAD (GADA) as a single antibody; the prevalence of positivity for autoantibodies to IA-2 (IA-2A) and ZnT8 (ZnT8A) was low in all age groups. Principal component analysis showed that the Amhara genomes were distinct from modern European and other African genomes. HLA-DRB1*03:01 (*p* = 0.0014) and HLA-DRB1*04 (*p* = 0.0001) were positively associated with this form of diabetes, while HLA-DRB1*15 was protective (*p* < 0.0001). The mean type 1 diabetes genetic risk score (derived from European data) was higher in patients than control participants (*p* = 1.60 × 10^−7^). Interestingly, despite the modest sample size, autoantibody-positive patients revealed evidence of association with SNPs in the well-characterised MHC region, already known to explain half of type 1 diabetes heritability in Europeans.

**Conclusions/interpretation:**

The majority of patients with insulin-dependent diabetes in rural North-West Ethiopia have the immunogenetic characteristics of autoimmune type 1 diabetes. Phenotypic differences between type 1 diabetes in rural North-West Ethiopia and the industrialised world remain unexplained.

**Electronic supplementary material:**

The online version of this article (10.1007/s00125-020-05229-x) contains peer-reviewed but unedited supplementary material, which is available to authorised users.

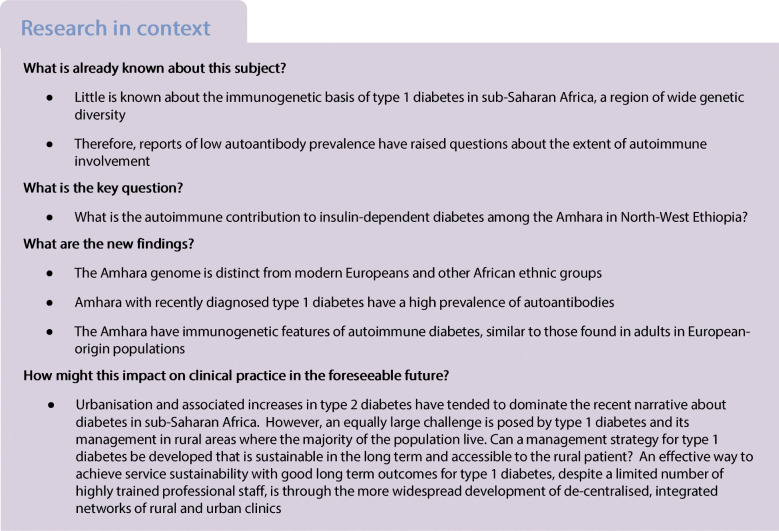



## Introduction

Type 1 diabetes is poorly characterised in many low- and middle-income countries of sub-Saharan Africa; specifically, there has been uncertainty about whether its pathogenesis is similar to the classic form of the disease found in industrialised countries. Striking phenotypic differences from the classic form of type 1 diabetes have been reported from several locations in sub-Saharan Africa; these include a low incidence in the pre-pubertal years with an age-specific peak in the third decade [1, 2], strong associations with low socioeconomic status [3], skewing of sex ratios with male predominance in some settings [4] and reported low autoantibody prevalence, suggesting a diminished role for autoimmune mechanisms in its aetiology compared with classic type 1 diabetes [5–7].

Classic type 1 diabetes is an autoimmune disease resulting from the interaction between genetic susceptibility [8] and the environment. Many loci have been shown to confer risk, of which the HLA class II genes remain the most important. Although the vast genetic diversity of Africa is well known [9, 10], very little is known about how, or if, this alters the genetic risk for type 1 diabetes in this region. Non-genetic, environmental factors also contribute to the pathogenesis of type 1 diabetes, their important role being inferred from the low concordance rate for the disease in identical twins, often less than 50% [11]. Environmental factors range from a putative role of viral infections in pancreatic beta cell death, see the review by Op de Beeck and Eizirik [12], to nutritional factors that may interact with genetic susceptibilities to determine disease risk [13]. There is a significant body of experimental evidence showing that moderate to severe, life-long undernutrition starting in utero affects pancreatic development and function by, inter alia, silencing key growth and differentiation factors [14–16], and by having an effect on the immune system [17]. Ethiopia is an example of a country that has a long history of repeated famines, and approximately 40% of children have evidence of nutritional stunting [18]. In these communities, undernutrition starts in utero and continues throughout life. The widespread experience of undernutrition in some areas of sub-Saharan Africa, taken in conjunction with the low levels of islet-cell autoimmunity in many historic reports from Africa [5–7], has raised the possibility that insulin-dependent diabetes in these populations may have a nutritional origin. Those with the lowest BMIs (15–16 kg/m^2^) were said to have ‘protein-deficient pancreatic diabetes’ [19], a type of malnutrition-related diabetes which has since been removed from WHO classifications. So, although there is agreement that environmental factors are important, it is not easy to prove their aetiological significance in type 1 diabetes, probably because genetic factors are stable and environmental factors change with time.

On account of uncertainties about the role of autoimmunity in the pathogenesis of type 1 diabetes in sub-Saharan Africa, we have carried out an immunogenetic investigation in a consecutive, unselected cohort of newly diagnosed insulin-dependent diabetic participants from a community in Ethiopia. The genetic objectives were to investigate the genome-wide underpinnings of type 1 diabetes and the leading European type 1 diabetes risk loci in this population. The study was carried out in a rural community, the Amhara of North-West Ethiopia, whose socioeconomic conditions are typical of many regions in sub-Saharan Africa.

## Methods

### Setting

The study was based in Gondar, Ethiopia, a university city 750 km north-west of the capital, Addis Ababa. Gondar has a central university hospital and ten stable satellite/peripheral health centres, which provide care for all patients with diabetes in a predominantly rural health zone comprising 2.6 million people. In order for the study to be representative of both the rural and urban populations, the region chosen had to have a stable healthcare infrastructure that was inclusive of the 90% of the population who live in rural areas. The entire diabetes service in this region has been developed and overseen by the same consultant physician for more than 30 years; thus, diagnosis and treatment at both rural and urban clinics have been carried out under the oversight of the same clinical team with the same treatment and management plan. Epidemiological studies of the diabetes care and outcomes in this region have been extensively described [1, 4, 20].

### Participants

Patients were entered sequentially into this study without selection bias. All patients and control participants were from the Amhara, the second-largest ethnic group in Ethiopia (approximately 29% of the total Ethiopian population); their language is Amharic, a Semitic language in the Afroasian group of languages [21] and, phenotypically, the Amhara have many Caucasoid features. This report includes data from patients aged up to 35 years with insulin-requiring diabetes and attending the University of Gondar Diabetes and Paediatric Clinics and associated rural clinics. All patients presented with a high plasma glucose and a degree of metabolic decompensation; all complained of weight loss (or parents worried about a very sick child), polydipsia and polyuria; most of the children and a high percentage of adults (50%) were ketoacidotic at presentation. All patients included in the study required insulin treatment continuously from first presentation. Control participants were hospital attendees selected in the same manner as previously [3]; eligibility criteria for control participants included no history of diabetes, normal random plasma glucose, aged up to 35 years and belonging to the Amhara ethnic group. Control samples were used only for autoantibody, HLA and genome investigations.

### Clinical characterisation

After metabolic stabilisation, a questionnaire was verbally administered in Amharic to record details of the duration and treatment of their diabetes, and details of their education, occupation and socioeconomic circumstances; height and weight were measured as previously described [3]. BMI was calculated as weight (kg) divided by height squared (m^2^). Age and sex-adjusted height and BMI *z* scores were derived using WHO Anthro software (version 3.2.2; https://www.who.int/growthref/tools/en/; accessed 15 June 2019) [22]. Whole body bioimpedance was measured using a Bodystat meter (Bodystat, Douglas, Isle of Man) and fat mass was calculated by the method of Kotler et al [23]. Venous blood samples for study purposes were obtained at a median of 2.5 months (IQR 1–7) from diagnosis using vacutainers, and plasma separation was carried out on site; samples were stored at −70°C, initially in Gondar and subsequently in the UK.

### Laboratory analyses

Autoantibodies to GAD (GADA), IA-2 (IA-2A) and ZnT8 (ZnT8A) were measured by radiobinding assays as previously described [24, 25]. All samples found to be GADA-positive using full-length GAD65 were re-assayed using truncated GAD_65_(96–585) radiolabel [26]; whereas 17 (8.5%) control participants were positive for GADA in the full-length GAD assay, the number were reduced to four (2.0%) in the truncated GAD assay. As only 6 of 137 patients found positive for GADA with full-length GAD were negative in the truncated GADA assay, results for truncated GAD_65_(96–585) were used in subsequent analyses. C-peptide was measured by Roche ‘ECLIA’ C-peptide chemiluminescence assay on a Cobas 8000 E602 machine (Switzerland), with a minimal detection limit of 0.010 μg/l.

### Genotyping and quality control

After extraction of DNA, patients and control participants were genotyped on the Infinium OmniExpress Exome Beadchip platform (Illumina, San Diego, CA, USA) at the Children’s Hospital of Philadelphia Center for Applied Genomics (Philadelphia, PA, USA). Quality control was performed using PLINK (v.1.90Beta4.5) [27], excluding individuals with discordant sex information, duplicate individuals and individuals with missing genotype >5%. SNPs with a call rate <95%, minor allele frequency <1% and Hardy–Weinberg equilibrium *p* < 10^−5^ were removed (708,143 SNPs remained).

HLA *DR* selected genotypes were measured by the method of Bunce et al [28]; due to the remaining limited volume of extracted DNA, 188 of 236 patients and 152 of 200 control participants were HLA typed.

We carried out a genome-wide association study (GWAS), a genetic risk score (GRS) analysis for type 1 diabetes and principal component (PC) analyses: details are in the Statistical analysis section (below).

### Statistical analysis

Anthropometric, metabolic, autoantibody and HLA statuses of patients were tested by *t* tests or ANOVA for continuously distributed variables (using log transformation where appropriate) or by χ^2^ tests for discrete variables. A *p* value of <0.05 was considered to be statistically significant.

GWAS was carried out using a univariate linear mixed model within GEMMA (https://github.com/genetics-statistics/GEMMA v. 0.94) [29], which accounts for population stratification and relatedness using the Wald test. Additionally, 55 established type 1 diabetes-implicated signals and their proxy SNPs were tested (11,748 SNPs) [30], and 403 established type 2 diabetes-implicated variants and their proxy SNPs were also tested (24,926 SNPs) [31]. Proxy SNPs were found using raggr (http://raggr.usc.edu, v. 3.5.0, accessed 26 July 2018), with a linkage disequilibrium threshold of *r*^2^ < 0.8 in the European and African populations. The association tests were also performed in a restricted set of cases positive for at least one autoantibody.

GRS for type 1 diabetes was calculated using PLINK by multiplying the number of risk-increasing alleles by the natural log of the OR at each locus and summing the OR across risk loci for each individual. Included were 19 SNPs (electronic supplementary material [ESM] Table 1) in the GRS, using weights as previously described [32]. The distribution of the type 1 diabetes GRS was compared for all diabetes cases vs control participants and for autoantibody-positive cases using linear regression, adjusting for sex and the first four PCs.

PC analysis was performed using PLINK as follows: the 1000 Genomes [33] and Ethiopian genotype files were merged, removing 4277 SNPs with location conflicts. SNPs with a minor allele frequency <0.01 were removed, and linkage disequilibrium pruning was performed at *r*^2^ < 0.2 between any two SNPs. Also removed was one individual from each pair with an identity-by-descent value >0.3 (46 individuals removed). PLINK was then used to calculate PCs and R (v.3.5.0) was used to plot the first three PCs.

### Ethical approval

The study was approved by the institutional ethics review boards of Gondar College of Medicine and Health Sciences and the UK National Research Ethics Services Committee (REC; reference: 14/WA/0132). Written, informed consent was obtained from all participants or their parents, as appropriate.

## Results

The characteristics of the diabetes cases are shown in Table 1; results for antibody positivity in control participants are shown separately below the patient results. Patients were investigated at a median of 2.5 months after clinical diagnosis. The overwhelming majority (89.4%) of the participants were born in rural areas around Gondar, Ethiopia. The median age at diagnosis was 21 years; only 36 (15.3%) were diagnosed at 15 years of age or younger, and most cases were in the 16–25 year age group. There was a striking male preponderance, but only in the post-pubertal age groups: 72.6% in the 16–25 year age group and 77.6% in the 26–35 year age group. The mean BMI SD *z* scores were lower than WHO norms in all age groups; in the adult groups, the BMIs (mean [SD]) were equivalent to 18.6 (2.4) and 19.8 (2.9) kg/m^2^ in the 16–25 and 26–35 year age groups, respectively. The percentage of body fat was also low in all age groups. All cases had low, but detectable, non-fasting C-peptide levels. The insulin treatment doses were <1 U/kg/day for all age groups. Of the 236 cases, 112 (47.5%) reported that their families’ source of income was subsistence farming or labouring, while only 41 (17.4%) had paid employment or owned businesses. Educational levels were low, with only 74 of the 200 adults (37%) reporting completed secondary education (not shown).

**Table 1:** Metabolic characteristics and autoantibody status at presentation of Ethiopian patients with type 1 diabetes

Characteristic	Age at onset (year)				*p* value
	0–15	16–25	26–35	All	
Number tested	36	124	76	236	
Male, *n* (%)	16 (44.4)	90 (72.6)	59 (77.6)	165 (69.9)	0.001
Blood glucose at diagnosis, mmol/l, median (IQR)	29.3 (27.1–33.3)	29.7 (24.0–33.3)	26.1 (20.4–32.2)	28.3 (22.2–33.3)	0.03
Insulin dose after stabilisation, U/kg/day, mean (SD)	0.92 (0.37)	0.79 (0.23)	0.66 (0.18)	0.77 (0.26)	<0.001
Diabetes duration, months, median (IQR)	3 (1–7)	2 (1–6)	2 (1–7)	2.5 (1–7)	NS
C-peptide, μg/l, median (IQR)	0.46 (0.32–1.09)	0.77 (0.33–1.35)	0.98 (0.46–1.87)	0.80 (0.34–1.42)	0.03
Height, SD *z* score, mean (SD)^a^	−1.49 (1.09)	−1.18 (0.91)	−1.09 (0.83)	−1.20 (0.92)	NS
BMI, SD *z* score, mean (SD)^a^	−1.20 (1.14)	−1.44 (1.11)	−0.97 (1.16)	−1.25 (1.15)	0.02
% body fat, mean (SD)	6.2 (8.7)	11.9 (7.7)	13.7 (6.5)		<0.001
Rural birth, *n* (%)	30 (83.3)	115 (92.7)	66 (86.8)	211 (89.4)	NS
Autoantibody prevalence, *n* (%)^b^					
GADA^c^	29 (80.6)	69 (55.6)	33 (43.4)	131 (55.5)	0.001
IA-2A	1 (2.8)	5 (4.0)	2 (2.6)	8 (3.4)	NS
ZnT8A	6 (16.7)	11 (8.9)	7 (9.2)	24 (10.2)	NS
Any antibody present	31 (86.1)	74 (59.7)	38 (50.0)	143 (60.6)	0.001

^a^Based on WHO standards, see Methods

^b^Note: Non-diabetic controls, *n* = 200: positive for autoantibodies, GADA, *n* = 4; IA-2A, *n* = 2; ZnT8A, *n* = 5; any antibody, *n* = 11

^c^GADA, antibodies against truncated GAD, i.e. GAD_65_(96–585)

### Autoantibody status

Table 1 shows that 86.1% of cases in the 0–15 year age group, 59.7% of cases in the 16–25 year age group and 50.0% of cases in the 26–35 year age group were autoantibody positive. GADA was the most common autoantibody and the most common when there was a single autoantibody; thus, 114 of 143 autoantibody-positive cases had GADA as the only autoantibody. Of control participants, 2% were GADA positive. The prevalence of GADA declined from 80.6% in those aged ≤15 years to 55.6% in the 16–25 year age group and 43.4% in the 26–35 year age group. In contrast, the prevalence rates of ZnT8A and IA-2A were low, even in those with childhood onset, without any age-specific trend. The GADA-positive and GADA-negative groups were further compared (ESM Table 2). In comparison with the autoantibody (GADA)-positive group, the negative (GADA) group were slightly older (23.6 vs 20.4 years, *p* < 0.001), had lower plasma glucose at presentation (26.7 vs 29.8 mmol/l, *p* = 0.01), had higher C-peptide levels (0.96 vs 0.71 μg/l, *p* = 0.015) and had lower insulin requirements (0.72 vs 0.81 U/kg, *p* = 0.012). They had similar BMIs (SD *z* score −1.25, or 19.3 kg/m^2^ [mean for both]). Percentage body fat was not significantly different in the two groups: GADA negative, 12.7% vs GADA positive, 10.8%; *p* = 0.065.

### Genomic analysis

#### PC analysis

**Fig. 1:**
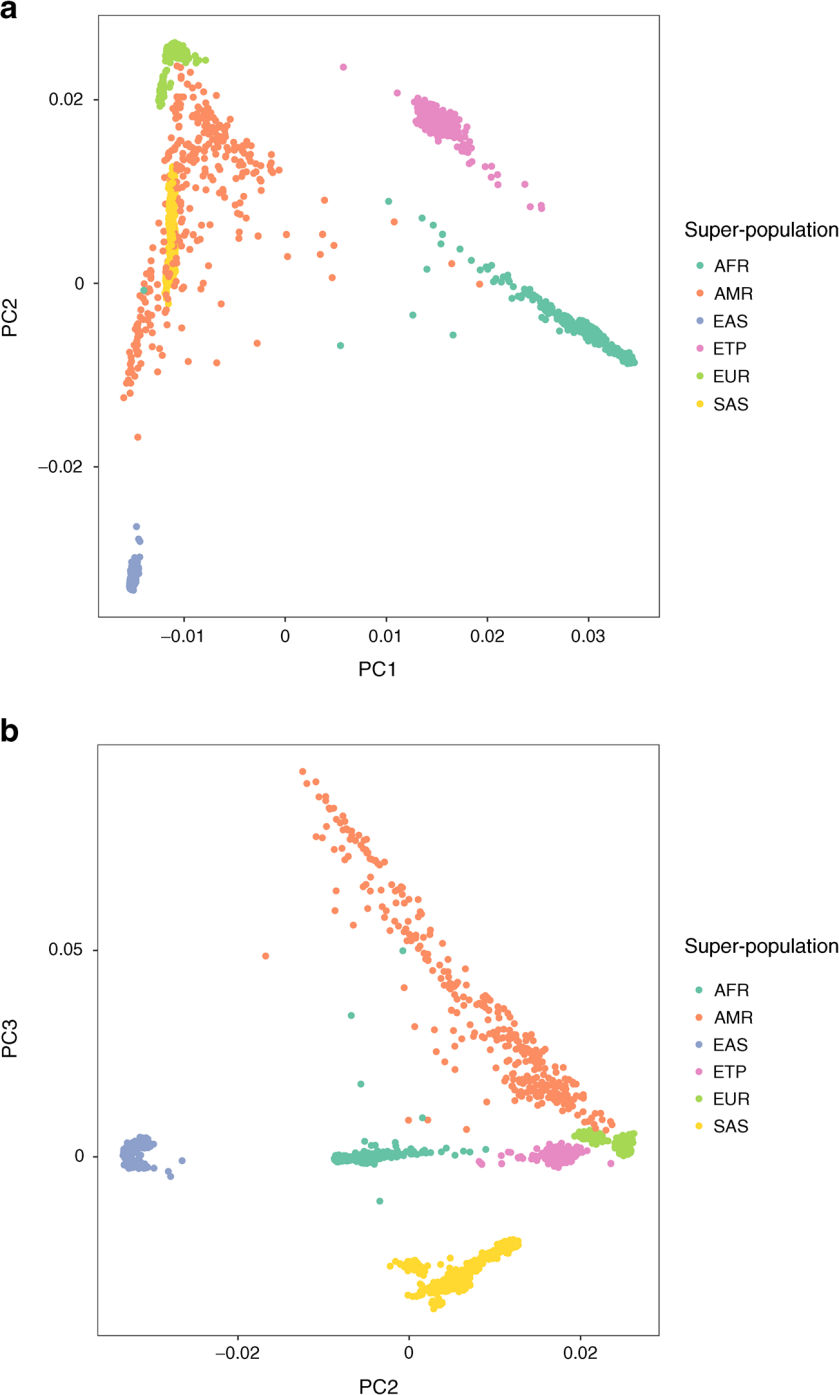
PC analysis. (**a**) The first two PCs (PCs 1 and 2). (**b**) The second and third PCs (PCs 2 and 3). These are based on genome-wide genotypes of the Ethiopian (Amhara) participants compared with 1000 Genomes ancestral groups. AFR, African; AMR, Americas. EAS, East Asian. ETP, Ethiopian; EUR, European; SAS, Southeast Asian

PC analysis is a technique that identifies the major axes of variation in genetic data. Leveraging the genome-wide genotyping data, the first PCs were plotted against the 1000 Genomes reference set [33] to visualise the relationship of the Ethiopian samples against worldwide super-populations (Fig. 1a, b). Comparing PC1 with PC2 (Fig. 1a), a clear separation was observed of the Ethiopian sample from the 1000 Genomes groups, and plotting PC2 vs PC3 (Fig. 1b) revealed clearly that the sample from the Amhara of Ethiopia was distinct from European and other African populations.

#### HLA analysis

HLA-DRB1*03:01 was positively associated with diabetes (81/188, 43.1%) compared with control participants (40/152, 26.3%; OR 2.12; *p* = 0.0014), and this association persisted when considering only GADA-positive cases (54/102, 52.9%) (Table 2). HLA-DRB1*04 was also positively associated with diabetes (83/188, 44.1%) vs control participants (37/152, 24.3%; OR 2.46; *p* = 0.0001); this association persisted when GADA-positive cases only were considered (51/102, 50.0%). HLA-DRB1*15 was strongly protective in the total diabetes group (7/188, 3.7%) compared with control participants (25/152, 16.4%; OR 0.20; *p* < 0.0001); this association persisted when GADA-positive cases only were considered (2/102, 2.0%). In GADA-negative cases, these three HLA disease associations were less striking (HLA-DRB1*03:01 [31.4%, *p* = 0.40])*,* but, importantly, with significant risk with HLA-DRB1*04 (37.2%, *p* = 0.035) and significant protection with HLA-DRB1*15 (5.8%, *p* = 0.018).

**Table 2:** Analysis of the frequency of the three most prominent type 1 diabetes risk alleles in European-origin populations among Ethiopian patients with diabetes and non-diabetic controls

Allele	Patients with diabetes, *n* (%)			Controls *n* = 152	All diabetes vs controls		GADA-positive diabetes vs controls		GADA-negative diabetes vs controls	
	All *n* = 188	GADA positive *n* = 102	GADA negative *n* = 86							
					OR (95% CI)	*p* value	OR (95% CI)	*p* value	OR (95% CI)	*p* value
DRB1*03:01	81 (43.1)	54 (52.9)	27 (31.4)	40 (26.3)	2.12 (1.34–3.37)	0.0014	3.15 (1.85–5.35)	<0.0001	1.38 (0.78–2.44)	0.40
DRB1*04	83 (44.1)	51 (50.0)	32 (37.2)	37 (24.3)	2.46 (1.54–3.93)	0.0001	3.11 (1.82–5.32)	<0.0001	1.84 (1.04–3.27)	0.035
DRB1*15	7 (3.7)	2 (2.0)	5 (5.8)	25 (16.4)	0.20 (0.08–0.47)	<0.0001	0.10 (0.02–0.43)	0.0001	1.31 (0.12–0.85)	0.018

#### GRS

**Fig. 2:**
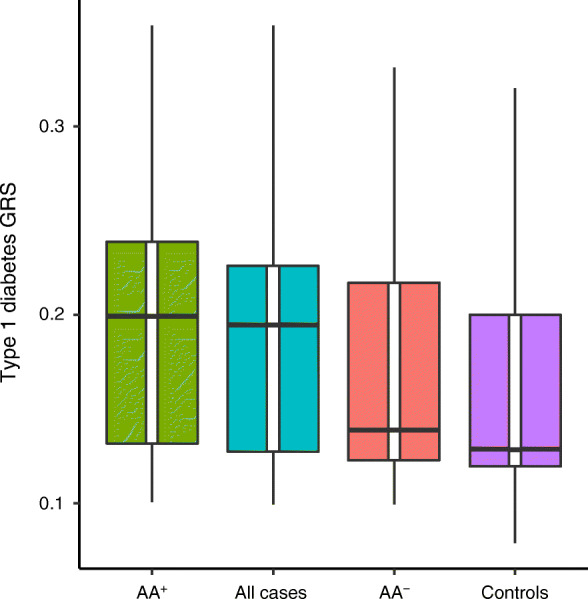
Box and whisker plot showing GRS for type 1 diabetes. The GRS was calculated using 19 established type 1 diabetes-associated SNPs of European background; variants, risk alleles and weights are listed in ESM Table 1 (see the Methods/Statistical analysis section for details). Diabetic patients were compared with control participants: autoantibody-positive cases (AA^+^) (*n* = 121, vs controls, *p* = 1.54 × 10^−9^), all cases (*n* = 187, vs controls, *p* = 1.6 × 10^−7^), autoantibody-negative cases (AA^−^) (*n* = 66, vs controls, *p* = NS) and control participants (*n* = 137). The vertical lines denote the maximum and minimum values

GRS for type 1 diabetes is shown in Fig. 2, and the type 1 diabetes-associated SNPs used in the GRS analysis are shown in ESM Table 1.

The average type 1 diabetes GRS was significantly higher for diabetes cases than for control participants. The mean (SD), compared with that of the control participants of 0.154 (0.067), was 0.189 (0.064) for the total diabetes group, 0.199 (0.067) for the autoantibody-positive group and 0.171 (0.057) for the autoantibody-negative group, with *p* = 1.6 × 10^−7^, *p* = 1.54 × 10^−9^ and *p* = NS, respectively.

#### GWAS

(ESM Figs 1, 2, 3, Table 3; ESM Tables 3, 4) No single SNP achieved genome-wide significance (*p* < 5 × 10^−8^) when all diabetic participants were investigated as a single group, irrespective of autoantibody positivity (ESM Figs 1 and 2). However, despite the modest sample size, SNPs within the HLA region were border-line GWAS significant (*p* < 5 × 10^−6^) for autoantibody-positive patients (Table 3, ESM Fig. 2), with the strongest signal falling in the *HLA-DQB1* locus (rs9273363, *p* = 5.13 × 10^−8^; Table 3). We also observed suggestively associated signals on chromosomes 4, 16 and 3 (Table 3). The GWAS analysis did not show genomic inflation (*λ* = 1.01; ESM Fig. 3). We then took a candidate SNP approach and extracted all type 1 and type 2 diabetes-associated SNPs; the strongest type 1 diabetes-associated signal was in the *HLA-DQB1* region (rs1063355, *p* = 6.28 × 10^−6^) (ESM Table 3); however, no type 2 diabetes-associated loci achieved significance (ESM Table 4).

**Table 3:** GWAS: border-line genome-wide significant signals associated with autoantibody-positive diabetes cases (*p* < 5 × 10^−6^)

SNP	Chromosome	Position^a^	Minor/major allele	MAF^b^ in cases	MAF in controls	OR	CI	*p*	Locus
rs9273363	6	32626272	A/C	0.445	0.217	1.281	1.226–1.339	5.13 × 10^−8^	*HLA-DQB1*
rs2760985	6	32566398	A/G	0.261	0.088	1.363	1.288–1.443	1.16 × 10^−7^	*HLA-DRB1*
rs9268528	6	32383108	G/A	0.407	0.358	1.261	1.208–1.316	1.57 × 10^−7^	*BTNL2*
rs9268542	6	32384721	G/A	0.407	0.365	1.256	1.203–1.312	3.23 × 10^−7^	*BTNL2*
rs11947273	4	92544404	T/C	0.336	0.445	0.811	0.778–0.846	1.25 × 10^−6^	*CCSER1*
rs2187818	6	32395568	G/T	0.360	0.427	0.805	0.770–0.841	1.48 × 10^−6^	*HLA-DRA*
rs9268585	6	32397403	T/G	0.360	0.427	0.805	0.770–0.841	1.48 × 10^−6^	*HLA-DRA*
rs4784939	16	58468211	T/C	0.309	0.146	1.280	1.216–1.347	2.43 × 10^−6^	*GINS3*
rs996482	3	20583674	T/C	0.318	0.172	0.817	0.782–0.853	4.06 × 10^−6^	*SGOL1*

^a^Base pair position reported for genome build 37

^b^MAF, minor allele frequency. Note that minor allele is the effect allele

Statistics: Linear mixed model with Wald test in GEMMA

## Discussion

This detailed study of consecutive, unselected, newly diagnosed patients in an impoverished, mainly rural population in sub-Saharan Africa shows that the majority have low C-peptide levels and low BMI, as well as diabetes-associated autoantibodies and diabetes risk alleles for type 1 diabetes; a smaller group are autoantibody negative and have some minor differences in their profile. As with previous studies in this and other locations in the region, the cases have a different disease phenotype; thus, the median age of onset is later than that observed in most industrialised countries and there is a striking male predominance in the post-pubertal age groups.

### Autoantibody prevalence

The prevalence of diabetes-related autoantibodies was high, falling from 86.1% in the youngest age group to 50.0% in the oldest group. This autoantibody frequency and age dependence were similar to [34, 35], if slightly lower [36] than, some other reports from industrialised countries, with all demonstrating lower prevalence of autoantibody positivity with increasing age of onset. However, whereas the majority of type 1 diabetes patients in industrialised countries have multiple diabetes-related autoantibodies, in this present Ethiopian study most had GADA alone, which was evident even in the 0–15 age group. Of note, the prevalence of autoantibodies to full-length GAD was relatively high in control participants, at 8.5% (mentioned in Methods section); however, in contrast to cases, the majority of these autoantibodies were directed to the low-risk N-terminal epitope [26]. The prevalence of other autoantibodies, IA-2A and ZnT8A, was low in all age groups, including those with childhood-onset disease. Our findings contrast with two urban studies from Ethiopia that found much lower levels of GADA, albeit with very low or absent IA-2A; however, these participants were studied 6 or more years after diagnosis [7, 37]. Unfortunately, comparisons in sub-Saharan Africa are difficult because there are very few studies where blood sampling for autoantibody status has taken place close to the time of diagnosis and involved both urban and rural populations, with the rural population forming the majority in most countries in this region. Moreover, the vast genetic diversity in this continent also complicates comparisons [9, 10, 38]. A recent study of type 1 diabetes from West Africa (Cameroon) investigated an urban group shortly after clinical diagnosis and compared them with a Belgian population. As in Ethiopia, few (9%) had childhood-onset type 1 diabetes, and in those who were classified as having type 1 diabetes a very low percentage of children (≤15 years) and adults (median age 30 years) were autoantibody positive; the majority of these had GADA, and far fewer had IA-2A or ZnT8A [2]: the overall autoantibody prevalence in those classified as having type 1 diabetes was lower than in the Amhara. The adult Belgian diabetic participants had a much higher incidence of multiple autoantibodies than the Cameroonian participants. The West Africans of Cameroon are of Bantu background and belong to a different genomic group to the Amhara [9, 10, 38]. These findings of different autoantibody profiles among ethnic groups are echoed by studies in South Africa comparing the profiles of type 1 diabetes in black (mixed ethnicity) and white participants. Interestingly, the peak age of clinical onset was later in black compared with white participants, and both groups were heavier than the Amhara (average BMI for black and white participants, 24.0 kg/m^2^ and 22.4 kg/m^2^, respectively). When studied several years after clinical diagnosis (mean duration >5 years), overall GADA positivity was 60% and 66% for black and white participants, respectively; IA-2A positivity was lower in the former than the latter (19% vs 41%, respectively), especially in those aged >21 years at diagnosis (7.3% vs 33%, respectively) [39]. Taken together with our data, these results [2, 39] from three different regions of sub-Saharan Africa suggest that there is considerable heterogeneity in the prevalence of autoantibody profiles both within Africa and between African and European populations. These differences appear to be independent of the age of onset of diabetes and, for the present, remain unexplained.

### Genomic analysis

Using genome-wide genotyping data, our study population was shown to be distinct from European and other African ancestral groups; based on what is known of their demographic and linguistic history, this was not unexpected [10]. GWAS and HLA analysis confirmed the predominant HLA association with type 1 diabetes-associated risk and protective alleles. By contrast, and of note, no associations were found for type 2 diabetes-associated loci. However, our sample size was low and power limited, and therefore the results should be seen as relatively preliminary. In view of the autoantibody and HLA results a GRS was applied, based on SNPs associated with type 1 diabetes on a European genetic background. This was done with the knowledge that the results might not be as robust when applied to a different ethnic group [40]. Despite the European background of the SNPs employed, the Ethiopian diabetes group showed a significantly increased GRS compared with control participants, mainly associated with autoantibody-positive cases. In addition, these Amhara diabetes patients showed an association with HLA class II alleles HLA-DRB1*03:01 and HLA-DRB1*04, in both the total diabetes cohort as well as in those with GADA, while HLA-DRB1*15 was strongly protective for diabetes. GADA-negative patients demonstrated an association with HLA-DRB1*04 and protection with HLA-DRB1*15. In short, they have some of the immunogenic features of the autoimmune diabetes of adolescent and adult European-origin and Chinese populations [41], while the diabetes-associated autoantibody, GADA, was similarly the dominant autoantibody and associated with HLA-DRB1*03:01, consistent with heterogeneity of type 1 diabetes endotypes [42, 43]. Our observations imply a widespread commonality in GADA-dominant, HLA-associated adult-onset autoimmune diabetes, despite global variation in the precise HLA-associated genotypes. Moreover, as with adult-onset autoimmune diabetes in Europe and China, both IA-2A and ZnT8A had lower frequencies and, in the present Amhara cohort, even in childhood-onset type 1 diabetes the prevalence of IA-2A was very low.

### Implications

Given that the majority of patients with diabetes in this rural Ethiopian population appear to have an autoimmune basis to their diabetes, the results do not support the hypothesis previously suggested by ourselves and others [1, 19] that the disease could have a direct relationship to undernutrition during prenatal and early postnatal life. Although type 1 diabetes is strongly associated with low socioeconomic status and skeletal disproportion in this community [3], it remains unclear whether nutrition or related aspects of poverty influence disease development and contribute to the relatively late peak age of onset of their diabetes. Of note, communities with diverse ethnic backgrounds in sub-Saharan Africa, but without (reported) malnutrition, have found a later peak age incidence [2, 39]. One line of evidence does, however, point to the involvement of a complex gene–environment interaction affecting the age of disease onset. Ethiopian Jews moved in large numbers from rural areas around Gondar to urban areas of Israel in the 1980s and 1990s. After immigration to Israel the age of onset of type 1 diabetes in the young offspring of these Ethiopian emigres slowly fell (in those with at least two high-risk alleles for diabetes), in proportion to the time that their Ethiopian-born parents had been resident in Israel; at the same time the incidence of type 1 diabetes in these young people rose dramatically, to be one of the highest within the Jewish communities of Israel [44].

Another unexplained phenotypic feature in our study was the marked male preponderance in the post-pubertal but not pre-pubertal age groups; this is not a feature of type 2 diabetes in Ethiopia [4]. A less marked post-pubertal male preponderance in type 1 diabetes has been noted previously in European cohorts [45, 46]. The cause of this striking sex bias is not clear, but may include sex-related immune and differential epigenome effects [17, 47].

### Limitations of the study

There is wide genetic diversity in sub-Saharan Africa; that being the case, we have studied a single ethnic group to limit genetic diversity within this study and have emphasised that the results from the Semitic-background Amhara differ in autoantibody profile from the Bantu-background Cameroonians [2], who were investigated at a similar time, close to clinical diagnosis. Additionally, both the Amhara and Bantu have shown some differences from European background groups. Our results, therefore, are not fully generalisable, neither when confined to sub-Saharan Africa nor on a more global basis. However, the differences associated with genetic diversity can be used as a tool to help throw light on the relationships between genetic background and autoantibody profile in type 1 diabetes.

In summary, the majority of insulin-dependent diabetes in the Amhara of North-West Ethiopia is autoimmune in nature, and the genetic risk and protective factors for type 1 diabetes are largely common to those found in Europeans with type 1 diabetes. The results of this study and reports from other areas of sub-Saharan Africa highlight the need for a wider understanding of the gene–environment interactions giving rise to differences in timing of peak incidence, male preponderance (in those with post-pubertal onset) and autoantibody profile in type 1 diabetes.

## Electronic supplementary material

ESM (PDF 352 kb)

## Data Availability

The datasets generated during the current study are available by application to the corresponding author on reasonable request.

## Abbreviations

GADA: Autoantibodies to GAD
GRS: Genetic risk score
GWAS: Genome-wide association study
IA-2A: Autoantibodies to IA-2
PC: Principal component
ZnT8A: Autoantibodies to ZnT8
