# Supplementary material for: Type 1 diabetes in Africa: an immunogenetic study in the Amhara of North-West Ethiopia
Source: Diabetologia. 2020 Jul 23;63(10):2158–68. doi: 10.1007/s00125-020-05229-x (PMC7476916; doi:10.1007/s00125-020-05229-x)

ESM Table 1.

Genetic Risk Score (GRS) calculation

- SNPs with established type 1 diabetes association used in GRS calculation (Oram et al, ref 32).

- Plus allele frequency in present study

Chromosome	SNP	Risk Allele	Odds Ratio	Weight	Allele frequency in present study (cases/ controls)
1	rs3024505	G	1.19	0.17	0.888/0.854
2	rs3087243	G	1.22	0.2	0.733/0.679
2	rs1990760	T	1.16	0.15	0.436/0.376
6	rs2395029	T	2.5	0.92	0.957/0.945
6	rs9388489	G	1.17	0.16	0.398/0.385
10	rs10509540	T	1.33	0.29	0.880/0.836
10	rs11594656	T	1.19	0.17	0.794/0.807
12	rs2292239	T	1.35	0.3	0.283/0.256
14	rs1465788	C	1.16	0.15	0.607/0.635
15	rs3825932	C	1.16	0.15	0.433/0.416
15	rs17574546	C	1.14	0.13	0.142/0.106
16	rs4788084	G	1.16	0.15	0.733/0.686
18	rs1893217	G	1.2	0.18	0.078/0.091
18	rs763361	T	1.16	0.15	0.677/0.657
19	rs425105	T	1.16	0.15	0.866/0.883
20	rs2281808	C	1.11	0.1	0.840/0.832
22	rs5753037	T	1.1	0.1	0.231/0.257
6	rs2187668, rs7454108	*DR3/DR4-DQ8*	48.18	3.87	rs2187668-T: 0.278/0.140 rs7454108-C: 0.099/0.055
6	rs2187668, rs7454108	*DR3/DR3*	21.12	3.05	rs2187668-T: 0.278/0.140 rs7454108-C: 0.099/0.055
6	rs2187668 ,rs7454108	*DR4-DQ8 /DR4-DQ8*	21.98	3.09	rs2187668-T: 0.278/0.140 rs7454108-C: 0.099/0.055
6	rs2187668, rs7454108	*DR4-DQ8/X*	7.03	1.95	rs2187668-T: 0.278/0.140 rs7454108-C: 0.099/0.055
6	rs2187668, rs7454108	*DR3/X*	4.53	1.51	rs2187668-T: 0.278/0.140 rs7454108-C: 0.099/0.055

ESM Table 2.

Comparison of the metabolic, anthropometric and phenotypic characteristics of the patients with diabetes, according to the presence or absence of GADA autoantibody

	Patients with diabetes		*p*-value
	GADA positive	GADA negative	
No of patients	131	105	
Age of onset (year), mean(SD)	20.4(6.1)	23.6(6.7)	<0.001
Male gender, n (%)	89(67.9)	76(72.4)	ns
Plasma glucose at diagnosis, mmol/l, median (IQR)	29.8(24.6-33.3)	26.7(20.9-33.3)	0.01
Insulin dose after stabilisation, units/kg, mean (SD)	0.81(0.27)	0.72(0.22)	0.012
Diabetes duration, months, median (IQR)	3(1-7)	2(1-6)	ns
C-peptide, µg/L, median (IQR)	0.71(0.31-1.26)	0.96(0.39-1.76)	0.015
Height, SD z score, mean (SD)	-1.17(0.98)	-1.23(0.83)	ns
BMI, SD z score, mean (SD)	-1.25(1.07)	-1.25(1.23)	ns
BMI(kg/m^2)[a]	19.3(2.3)	19.3(2.9)	ns
% body fat, mean(SD)	10.8(7.7)	12.7(7.9)	0.065
Rural Birth, n (%)	121(92.4)	90(85.7)	ns

[a]Adults ≥ 16 yrs.

ESM Table 3. **Type 1 diabetes-related signals in all cases with diabetes at an uncorrected *p*<0.05.**

No signal remained significant after multiple test correction ($p< 4.26 \times 10^{-6}$)

SNP	Locus	Chr	Position	Minor/Major Allele	MAF[a] in Cases	MAF in Controls	OR	CI	*p*-value
rs3024493	*IL10*	1	206943968	A/C	0.083	0.135	0.88	0.83-0.93	3.21×10^{-2}
rs1063355	*HLA-DQB1*	6	32627714	T/G	0.275	0.420	0.85	0.81-0.88	6.04×10^{-5}
rs4235991	*DLL1*	6	170382923	G/A	0.233	0.321	0.90	0.86-0.95	1.76×10^{-2}
rs667899	*PRKCQ*	10	6464156	A/G	0.294	0.201	1.13	1.07-1.18	9.96×10^{-3}
rs229541	*C1QTNF6*	22	37591318	G/A	0.401	0.329	1.10	1.05-1.14	3.06×10^{-2}

Type 1 diabetes-related signals in cases positive for at least one autoantibody at an uncorrected *p*<0.05.

No signal was significant after multiple test correction ($p<4.26 \times 10^{-6}$)

SNP	*Locus*	*Chr*	*Position*	*Minor/Major Allele*	*MAF in Cases*	*MAF in Controls*	*OR*	*CI*	*p-value*
rs1063355	*HLA-DQB1*	6	32627714	T/G	0.229	0.420	0.81	0.77- 0.85	6.28×10^{-6}
rs667899	*PRKCQ*	10	6464156	A/G	0.297	0.201	1.13	1.07-1.19	1.8×10^{-2}
rs11170466	*ITGB7*	12	53585859	T/C	0.131	0.069	1.17	1.09-1.26	2.7×10^{-2}
rs12161793	*CSAD*	12	53552475	G/A	0.161	0.095	1.15	1.08-1.23	3.2×10^{-2}
rs4235991	*WDR27*	6	170382923	G/A	0.241	0.321	0.91	0.86-0.95	4.7×10^{-2}

[a]MAF= minor allele frequency   Statistics: Linear mixed model with Wald test in GEMMA

ESM Table 4 **Type 2 diabetes-related signals in total diabetes group at an uncorrected *p*<0.05.**

No signal was significant after multiple test correction ($p<2.00 \times 10^{-6}$)

SNP	*Locus*	*Chr*	*Position*	*Major/Minor Allele*	*MAF[a] in Cases*	*MAF in Controls*	*OR*	*CI*	*p-value*
rs1493694	*NOTCH2*	1	120526982	T/C	39.6%	30.7%	1.12	1.07-1.16	7.71×10^{-3}
rs4402960	*IGF2BP2*	3	185511687	T/G	51.9%	44.5%	2.19	2.10-2.28	4.33×10^{-2}
rs6813195	*TMEM154*	4	153520475	T/C	39.6%	49.6%	0.90	0.87-0.94	8.64×10^{-3}
rs3129948	*BTNL2*	6	32354644	A/C	27.5%	18.3%	1.13	1.08-1.19	7.69×10^{-3}
rs2968553	*ADCK2*	7	140382156	T/C	43.9%	52.9%	0.45	0.43-0.47	4.27×10^{-2}
rs6476842	*GLIS3*	9	4291268	C/T	16.6%	23.4%	2.49	2.37-2.62	4.82×10^{-2}
rs4146894	*PLEKHA1*	10	124155381	C/T	46.5%	38.3%	2.27	2.18-2.36	4.15×10^{-2}
rs10400343	*HSD17B12*	11	43832505	G/A	27.8%	20.6%	2.56	2.46-2.67	3.84×10^{-2}
rs4275659	*ABCB9*	12	123447928	T/C	33.7%	47.5%	0.87	0.84-0.91	6.27×10^{-4}
rs1051434	*MPHOSPH9*	12	123641200	C/T	33.9%	48.9%	0.88	0.84-0.91	1.66×10^{-4}
rs1879379	*C12orf65*	12	123727443	G/A	35.3%	51.4%	0.85	0.82-0.89	5.75×10^{-5}
rs12970134	*MC4R*	18	57884750	G/A	18.7%	11.7%	1.12	1.07-1.18	2.52×10^{-2}

Type 2 diabetes-related signals in autoantibody positive cases at an uncorrected *p*<0.05.

No signal was significant after multiple test correction ($p<2.00 \times 10^{-6}$)

**SNP**	**Locus**	**Chr**	**Position**	**Minor/Major Allele**	**MAF in Cases**	**MAF in Controls**	**OR**	**CI**	***p-value***
rs35265698	*HLA-DRB1*	6	32561334	G/C	28.0%	12.5%	1.26	1.19-1.33	2.50×10^{-5}
rs984748	*RAB1A*	2	65369964	T/C	24.6%	40.5%	0.86	0.83-0.90	4.10×10^{-4}
rs4451914	*SIN3A*	15	75739824	C/T	49.6%	37.2%	1.17	1.12-1.22	6.82×10^{-4}
rs1051431	*MPHOSPH9*	12	123645803	A/G	41.1%	44.2%	0.86	0.83-0.90	9.64×10^{-4}
rs1493694	*NOTCH2*	1	120526982	T/C	43.2%	30.7%	1.15	1.10-1.20	1.78×10^{-3}
rs2937121	*WWP2*	16	69870409	G/T	30.5%	19.7%	1.17	1.11-1.23	1.81×10^{-3}
rs1035061	*PITPNC1*	17	65647063	G/A	27.1%	39.1%	0.87	0.83-0.91	3.47×10^{-3}
rs7559672	*ACVR2A*	2	147855299	C/T	10.6%	4.0%	1.27	1.17-1.38	3.58×10^{-3}

ESM Table 4 contd.									
SNP	Locus	Chr	Position	Minor/major Allele	MAF in cases	MAF in Controls	OR	CI	*p-value*
rs13212734	*RREB1*	6	7037637	A/G	21.2%	33.6%	0.87	0.83-0.91	$3.95x10^{-3}$
rs12778642	*HHEX*	10	94464307	T/G	21.2%	32.5%	0.88	0.84-0.92	$6.87x10^{-3}$
rs11068780	*WSB2*	12	118476079	T/C	4.2%	9.5%	0.80	0.73-0.87	$1.18x10^{-2}$
rs10276674	*DGKB*	7	14922007	C/T	36.9%	28.5%	1.13	1.07-1.18	$1.49x10^{-2}$
rs1296328	*PCDH18*	4	137083193	C/A	46.6%	36.0%	1.12	1.07-1.17	$1.50x10^{-2}$
rs1552224	*ARAP1*	11	72433098	C/A	4.6%	1.1%	1.40	1.21-1.60	$1.76x10^{-2}$
rs1358980	*VEGFA*	6	43764551	T/C	27.4%	36.5%	0.89	0.85-0.94	$1.79x10^{-2}$
rs4946812	*BEND3*	6	107431688	A/G	14.4%	21.9%	0.87	0.82-0.92	$1.89x10^{-2}$
rs10788575	*PTEN*	10	89768584	A/G	12.3%	6.6%	1.19	1.10-1.28	$1.95x10^{-2}$
rs12454712	*BCL2*	18	60845884	C/T	19.5%	28.8%	0.88	0.84-0.93	$1.99x10^{-2}$
rs11635472	*HERC1*	15	63975690	C/T	38.6%	47.1%	0.90	0.86-0.94	$2.16x10^{-2}$
rs11786992	*ESRP1*	8	95685147	C/A	33.9%	44.9%	0.91	0.87-0.95	$3.04x10^{-2}$
rs2303108	*ZC3H4*	19	47589895	T/C	42.4%	33.2%	1.10	1.05-1.15	$3.33x10^{-2}$
rs6575984	*MARK3*	14	103876454	A/G	47.5%	39.1%	1.10	1.05-1.15	$3.60x10^{-2}$
rs943005	*TFAP2B*	6	50865820	T/C	12.3%	18.6%	0.88	0.83-0.94	$3.74x10^{-2}$
rs7593685	*THADA*	2	43215855	C/T	30.3%	22.4%	1.11	1.06-1.17	$3.78x10^{-2}$
rs10803762	*ITGB6*	2	161105876	G/A	33.1%	42.7%	0.91	0.87-0.95	$3.91x10^{-2}$
rs10011174	*FBXW7*	4	153495515	G/A	35.6%	43.8%	0.91	0.87-0.95	$4.01x10^{-2}$
rs7124681	*CELF1*	11	47529947	A/C	36.0%	46.4%	0.92	0.88-0.96	$4.22x10^{-2}$
rs2183825	*LINGO2*	9	28412375	C/T	18.6%	25.9%	0.90	0.85-0.95	$4.41x10^{-2}$
rs13332406	*RBL2*	16	53489705	G/A	47.0%	36.9%	1.09	1.05-1.14	$4.69x10^{-2}$

[a]MAF= minor allele frequency Statistics: Linear mixed model with Wald test in GEMMA

ESM Fig 1. **Manhattan plot of GWAS results of all cases with diabetes vs controls**

Red line is genome-wide significance *p*-value threshold ($p < 5x10^{-8}$)
Blue line is line for suggestive significance (p<1x 10^{-5})

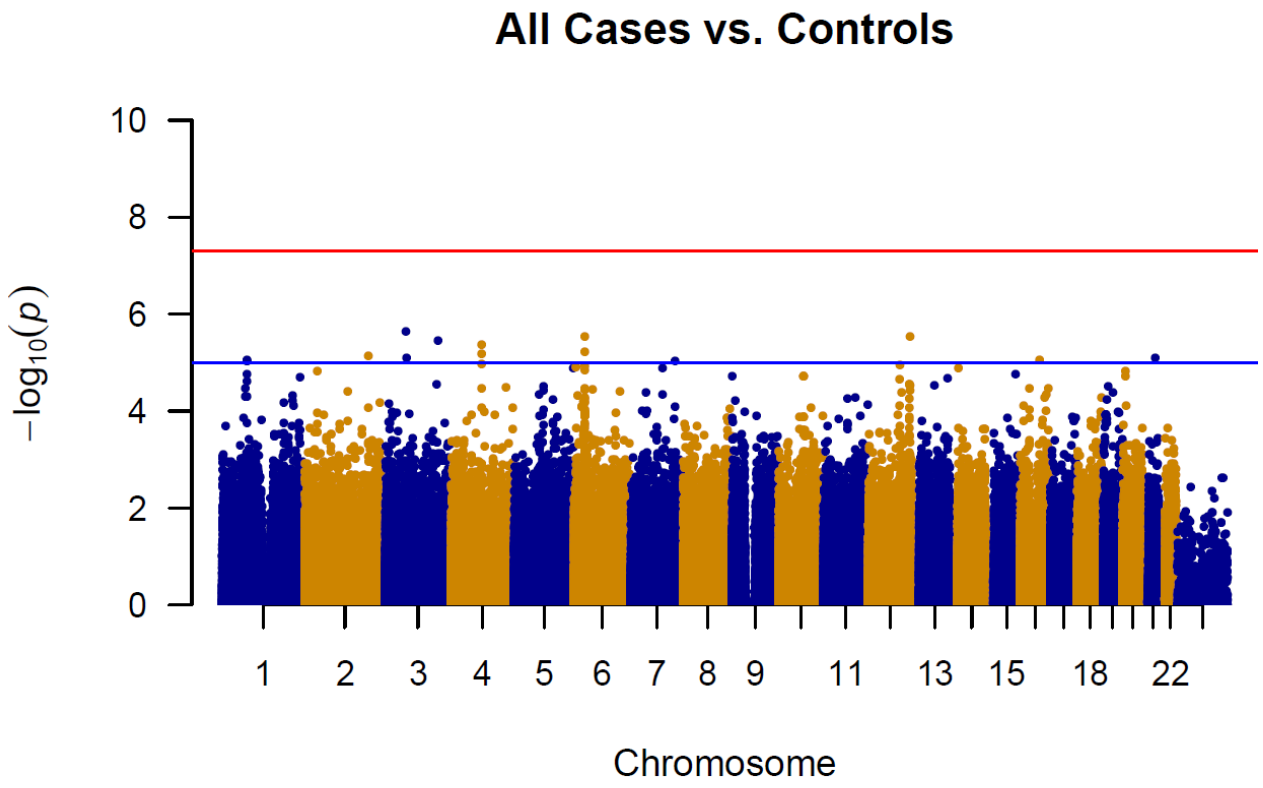

ESM Fig 2. **Manhattan plot of GWAS results for autoantibody positive (AA+) cases with diabetes vs controls**

Red line is genome-wide significance *p*-value threshold ($p < 5x10^{-8}$)
Blue line is line for suggestive significance ($p<1x\ 10^{-5}$)

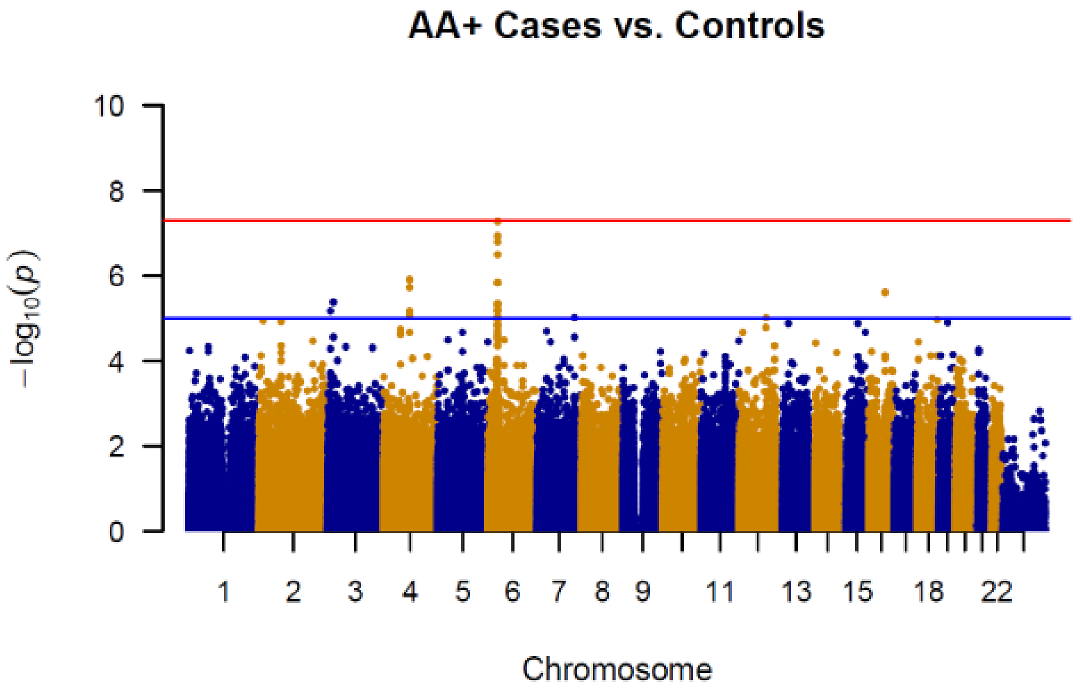

ESM Fig 3. **QQ plot of GWAS for autoantibody positive cases (AA+) with diabetes vs controls**

The QQ plot shows there is no genomic inflation, Lambda =1.01

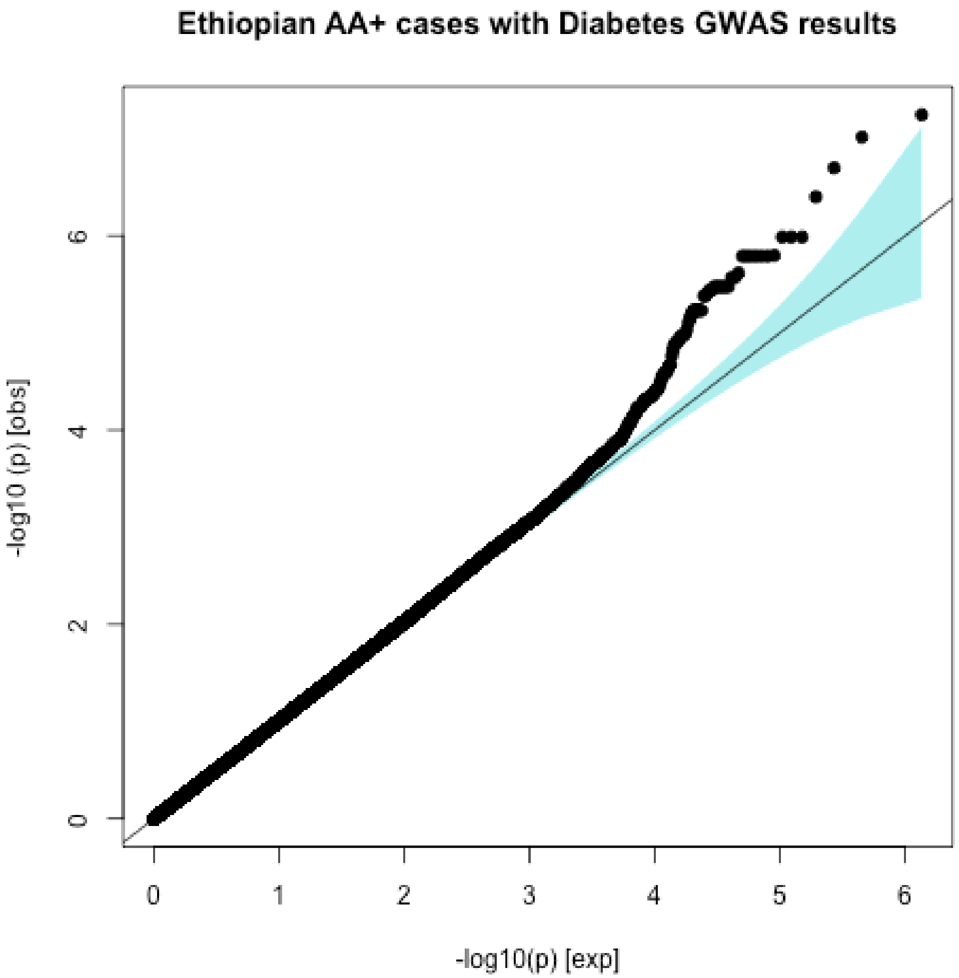

Supplement: Supplementary file 1 — (PDF 352 kb) [file 125_2020_5229_MOESM1_ESM.pdf]
